# Emergency department physicians’ distribution of time in the fast paced-workflow-a novel time-motion study of drug-related activities

**DOI:** 10.1007/s11096-021-01364-6

**Published:** 2021-12-23

**Authors:** Lisbeth D. Nymoen, Therese Tran, Scott R. Walter, Elin C. Lehnbom, Ingrid K. Tunestveit, Erik Øie, Kirsten K. Viktil

**Affiliations:** 1 Diakonhjemmet Hospital Pharmacy AS, Postbox 40 Vinderen, 0319 Oslo, Norway; 2 Department of Pharmacy, University of Oslo, Oslo, Norway; 3 Centre for Health Systems and Safety Research, Australian Institute of Health Innovation, Macquarie University, Sydney, Australia; 4 Department of Pharmacy, UiT The Arctic University of Norway, Tromsø, Norway; 5 Department of Health and Caring Sciences, Linnaeus University, Kalmar, Sweden; 6 Department of Internal Medicine, Diakonhjemmet Hospital, Oslo, Norway

**Keywords:** Emergency service hospital, Medication reconciliation, Medication errors, Practice management medical, Time and motion studies, Time management

## Abstract

*Background* In the emergency department physicians are forced to distribute their time to ensure that all admitted patients receive appropriate emergency care. Previous studies have raised concerns about medication discrepancies in patient’s drug lists at admission to the emergency department. Thus, it is important to study how emergency department physicians distribute their time, to highlight where workflow redesign can be needed.

*Aim* to quantify how emergency department physicians distribute their time between various task categories, with particular focus on drug-related tasks.

*Method* Direct observation, time-motion study of emergency department physicians at Diakonhjemmet Hospital, Oslo, Norway. Physicians’ activities were categorized in discrete categories and data were collected with the validated method of Work Observation Method By Activity Timing between October 2018 to January 2019. Bootstrap analysis determined 95% confidence intervals for proportions and interruption rates.

*Results* During the observation time of 91.4 h, 31 emergency department physicians were observed. In total, physicians spent majority of their time gathering information (36.5%), communicating (26.3%), and documenting (24.2%). Further, physicians spent 17.8% (95% CI 16.8%, 19.3%) of their time on drug-related tasks. On average, physicians spent 7.8 min (95% CI 7.2, 8.6) per hour to obtain and document patients’ drug lists.

*Conclusion* Emergency department physicians are required to conduct numerous essential tasks and distributes a minor proportion of their time on drug-related tasks. More efficient information flow regarding drugs should be facilitated at transitions of care. The presence of healthcare personnel dedicated to obtaining drug lists in the emergency department should be considered.

**Supplementary Information:**

The online version contains supplementary material available at 10.1007/s11096-021-01364-6.

**Impacts on practice**

- Physicians spend under eight minutes per hour on average to obtain and document patients’ drug lists at admission. This must be taken into consideration when using these lists as basis for further drug treatment during the hospital stay.
- This study has provided baseline data which is required to evaluate future quality improvements and work efficiencies regarding drug-related tasks conducted by emergency department physicians.
- This study highlights a need for a more seamless drug information flow for patients admitted to hospital.

## Introduction

Crowding is an increasing challenge in the fast-paced workflow of the emergency department (ED) [1]. Physicians are forced to distribute their time to ensure that all admitted patients receive adequate emergency care. In several countries, obtaining and documenting patients’ medication histories at ED admission are tasks assigned to ED physicians [2–7]. However, there is concerning evidence that approximately 60% of patients are registered with an incorrect drug list on admission [5, 8, 9]. And further, it has been indicated that obtaining drugs-lists is down-prioritized by physicians when the ED is crowded [7], Around half of the medication errors identified in hospitals occur on admission or at discharge [10], and up to 27% of hospital prescribing errors can be linked to inaccurate or incomplete ED drug lists obtained at admission [11]. Several studies have reported that dedicated personnel, such as pharmacists or pharmacy technicians obtain more complete and accurate drug lists in the ED setting compared to physicians [2–4].

Work tasks performed by ED physicians have been investigated in previous time-motion studies; however, these have focussed on length of stay, communication patterns, interruptions, multitasking, and time dedicated to direct patient care [12, 13]. There is a lack of studies focusing on what drug-related tasks ED physicians’ conduct and their time distribution between drug-related and non-drug-related tasks. As essential drug-related tasks at ED admission are assigned to physicians in several countries, it is important to investigate their work patterns, to highlight where workflow redesign is needed to improve patient safety regarding for instance medication discrepancies.

## Aim

The aim of this study was to quantify how ED physicians distributed their time between various task categories, with particular focus on the time spent on drug-related activities.

## Ethics approval

The study protocol was approved by the institutional review board. The Regional Committee for Medical and Health Research Ethics (Reference number: 2015/1356/REK South-East A) approved the study protocol August 8, 2018. Written informed consent was obtained from all participating physicians before inclusion.

## Method

### Study design

A continuous observational time-motion study of physicians in the ED at Diakonhjemmet Hospital (non-academic, urban), Oslo, Norway. The study was designed and reported according to the “Suggested Time And Motion Procedures (STAMP)” guidelines [14] and the STROBE statement [15]. Two observers (LDN -experienced clinical pharmacist, TT -pharmacy master student) performed direct observations between October 16, 2018, to January 8, 2019.

The validated method of Work Observation Method By Activity Timing (WOMBAT) [16, 17] was used to collect data. WOMBAT was developed to provide a reliable method for investigating the complexity of clinical work patterns. The method enables recording of multiple dimensions (*what, where, how*, and with *whom*) simultaneously, interruptions and multitasking, and thus chosen as the method in this study. Data were collected using a Samsung Galaxy 8 tablet running version 2 of the licenced WOMBAT software [16, 17].

### Study setting

In Norway patients are referred to the ED from healthcare personnel in the primary healthcare service e.g., general practitioner (GP) and municipal emergency clinic. The referring healthcare personnel set a tentative referral reason after assessing the patient’s symptoms and conducting an initial examination (before the ED admission). Every year around 14,000 patients are referred to the ED at Diakonhjemmet Hospital. The average length of stay in the ED is 3.2 h (2018). During this time physicians decide if the patient needs to be hospitalized or not. Emergency Medicine (EM) was first established as a physician speciality in Norway in 2017 and there are few EM specialists in Norway. Hence, physicians working at the ED, Diakonhjemmet Hospital are physicians from other specialities, rostered to cover shifts in the ED. Based on the tentative referral reason patients are allocated to see a physician from the Department of Internal Medicine (Medical physicians), or a physician from the Department of Surgery (Surgical physicians) at admission to the investigated ED. Medical physicians handle approximately 70% of referred patients and Surgical physicians handles 30% of patients.

In addition to physicians, the clinical ED staff at Diakonhjemmet Hospital consists of nurses triaging patients at arrival to the ED, taking measurements (e.g., blood pressure, temperature, echocardiography), monitoring symptoms, preparing, and administering drugs. A secretary handles administrative matters such as payment for foreign patients, obtaining discharge notes from earlier hospital stay at other hospitals or drug lists from GPs. Clinical pharmacists cover a 0.5 full-time equivalent pharmacist position (approximately 19 h per week) in the ED and primarily conducts medication reconciliation. When clinical pharmacists have conducted a medication reconciliation, the ED physician responsible for the patient is alerted. The physician utilizes the information obtained through medication reconciliation when taking medication history, and further document the drug list in the medication chart. Due to the limited pharmacist-coverage, majority of medication histories is obtained by physicians without pharmacists conducting medication reconciliation.

### Study population and sample size

During the data collection period 4-6 physicians were present in the ED at all times, 3-4 Medical physicians, and 1-2 Surgical physicians. Due to the roster-based affiliation of physicians to the ED the physician staff shifted frequently, hence inclusion and randomization of physicians were conducted consecutively before each observation session. All physicians present in the ED at the pre-set observation session time were eligible for inclusion. The observers randomized (by draw) which of the available physicians to observe. First a draw of affiliation (3:1, medical or surgical, due to the skewed distribution of physicians present in the ED), further a draw of experience level (1:1, experienced or inexperienced). Affiliation (Medical or Surgical) and experience level (inexperienced: interns and junior residents; experienced: senior consultants) was recorded for all included physicians.

The number of observation hours was selected based on previous time- and motion studies where approximately 62-137 h of observations were recorded [12, 18–20]. According to the aim of this study 90 h of observations were considered sufficient to accurately describe physicians work pattern.

### Data collection

Once included, the physician was continuously observed for one session (two hours), where observers recorded all conducted tasks (automatically time stamped by the WOMBAT-software). Each physician was observed for a maximum of two sessions. Observation sessions were two hours long to minimize participant and observer fatigue. The sessions were conducted according to a time schedule set by the observers, to ensure the data collection covered all hours between 9:00 am to 9:00 pm (80% of patients admitted to the ED arrive within this timeframe), both weekdays and weekends. The observation sessions were independent of the length of stay for patients treated by the observed physicians.

No previous time-motion study of ED physicians has defined drug-related tasks separately, therefore the discrete categories used in this study were conceptualised and structured based on the findings from a pre-study period. In the pre-study period ED physicians were followed and all conducted activities were recorded in plain text, including tasks conducted (*what)*, the locations physicians were in when conducting the task (*where*), the tools they used to conduct the tasks (*how*) and other persons involved in the conducted tasks (*who*). Further the recorded text was grouped in discrete categories and structured under four dimensions (*what, where, how, and who*) in line with earlier studies [20, 21]. The identified task categories for the *what* dimension (Table 1) were reviewed by an experience clinical pharmacist (KKV) and a chief physician (EØ). Thereafter categories in all dimensions were tested and evaluated during a pilot study before data collection, to ensure that all physician tasks were covered by the conceptualised categories. A detailed overview of categories within *where, how* and *who* dimensions is presented in electronic supplementary material 1.

**Table 1:** Work task categories (***What***), subcategories, definitions, and examples. Drug-related: all conversations, reading or writing that included information about the patients’ drugs or drug use. Non-drug-related: all other conversations, activities, reading and writing. Where, how and with whom the observed physicians conducted tasks presented in the table, was specified by categories in *where, how* and *who* dimensions in the WOMBAT-tool (Electronic supplementary material 1)

Task category	Subcategories	Definition	Examples
Examination/ Treatment		Direct, physical examination/treatment of the patient^1^	Examination of patient Taking samples (e.g., fecal occult blood test, arterial blood gas) Relocating shoulder, suture a wound Monitoring patients’ symptoms
Gather information	Drug-related	Gather drug-related information related to patients/ patient treatment	Physician obtained information about patients’ medication history by talking directly or by telephone to patients, next of kin, other hospitals, reading on computer or paper referral letters.
	Non-drug-related	Gather non-drug-related information related to patients/ patient treatment	Physician obtained information about patients’ medical history by talking directly or by telephone to patients, next of kin, other hospitals, reading on computer or paper referral letters.
Documentation	Drug-related	Documentation of drug-related patient information	Physician documented drug-related information on paper or on computer Prescribing drugs in medical chart/ Prescription Intermediary
	Non-drug-related	Documentation of non-drug-related patient information	Physician documented non-drug-related information on paper or on computer
Professional communication	Drug-related	Professional communication with other healthcare personnel/ patients/ next of kin about drug-related matters relevant to patients’ treatment	Physician communicated direct or via telephone with other healthcare personnel about patient drug-related treatment Physician informed patient, next of kin about further drug-treatment
	Non-drug-related	Professional communication with other healthcare personnel/ patients/ next of kin about non-drug-related matters relevant to patients’ treatment	Physician communicated direct or via telephone with other healthcare personnel about patient-related matters Physician informed patient, next of kin about further non- drug-related treatment
Social	Professional	Professionally relevant activities or communication not directly linked to patient treatment/ information	Digital courses Reading procedures (not directly regarding patient treatment) Send professional e-mail
	Non-professional	Social activities or communication (not professionally relevant)	Personal phone calls/ texting/ e-mailing Bathroom breaks Meal break
Unknown		Activities that could not be observed	Physician treated a patient in an infection isolated room (droplet- or airborne infections)
Hygiene		Activities to prevent communicable diseases	Physician washed/ disinfected hands
Movement		Movement between locations (***Where***)	Physician walked between locations (where categories)
Outside emergency department		Activities conducted outside the defined area of the emergency department	Physician were called on to assist patient on hospital ward (left the emergency department)

^1^During observation time physicians did not administer drugs to patients, hence only non-drug-related treatment and examination were recorded

Interruptions, defined as stopping the current task to respond to an external stimulus (e.g., a telephone call), and multitasking, defined as performing two (or more) tasks simultaneously, were recorded with the WOMBAT software.

To test the observers’ agreement on data collection categories (all dimensions, and timestamping), inter-rater reliability testing (IRR) was performed. The observers followed the same physician and independently recorded data for three separate sessions of 30 min each, once before data collection and twice during the data collection period. The IRR observation data were analysed after each session using a multivariate chance-adjusted agreement method (the iota score^,^ a multivariate generalisation of Cohen’s kappa) [22, 23], applied to the data in the format of one second time windows. The average iota score was 0.76 (before data collection: 0.781, during data collection: 0.622 and 0.867), indicating substantial agreement [24] between observers.

Patients were not observed in this study, however the number of patients treated by the observed physicians was recorded. Patients were classified as “new” or “follow-up”. Patients were classified as new when no one had taken their medical history, including medication history, prior to when the observed physician met the patient. Patients were classified as follow-up if a medical history, including a medication history, had already been obtained when the observed physician met the patient.

### Data analysis

Proportions of total observation time were defined as the time spent on each task category, accounting for any multitasking, divided by the total observation time. Proportions specific for physician groups and specific drug-related and non-drug-related time were calculated similarly, although the denominators were group specific (considering any overlap in time due to multitasking). The field of analyzing proportions of continuous time measures are scarcely investigated, hence a bootstrapping approach was used to generate 95% confidence intervals (CIs) for the proportions and interruption rates. Monte Carlo testing was applied for comparing drug-related task time between different physician groups: medical vs. surgical physicians, experienced vs. inexperienced physicians, significance level 0.05. Both bootstrapping and Monte Carlo testing were chosen to avoid the reliance on parametric assumptions which were not met by this data.

Descriptive statistics comprised the number of registered tasks and observed total task time. Data preparation was conducted in Microsoft Office Excel. Data were analysed using the SAS system for Windows, version 9.4, and IBM SPSS software, version 25.

## Results

**Fig. 1:**
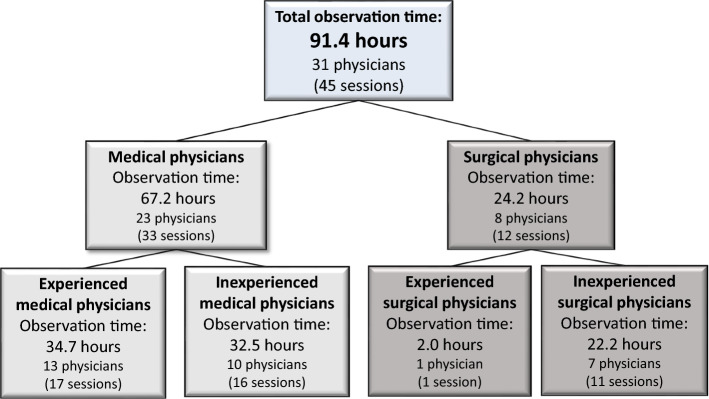
Distribution of included physicians. Observation time is reported as absolute observation time in hours. Experienced and inexperienced physicians were included from both Department of Internal Medicine (medical physicians) and Department of Surgery (surgical physicians)

A total of 31 physicians were observed to obtain a total observation time of 91.4 h, (Fig. 1), 14 of the physicians were observed for two sessions.

During each two-hour session, physicians attended to 2.7 patients on average (95% CI 2.3, 3.1). Of these, 2.0 patients (95% CI 1.6, 2.4) were new patients while 0.7 patients (95% CI 0.4, 1.0) were follow-up. Hence, physicians saw on average one new patient per hour in addition to follow-up patients.

Physicians spent 17.8% of their time conducting drug-related tasks and 83.3% conducting non-drug-related tasks (Table 2). Proportions add up to over 100% due to multitasking. Physicians multitasked for 17.4% (95% CI 14.8, 20.5) of the drug-related task time and 9.8% (95% CI 9.0, 10.7) of the non-drug-related task time (p<0.01).

**Table 2:** Physicians’ distribution of task time. Data on gathering information, documentation, and professional communication is specified by subcategories non-drug-related and drug-related

Task type	Number of recorded tasks	Observed total task time, hours	Proportion of time on task %^1, 2^ (95% CI)
Non-drug-related			83.3% (95% CI 80.0%, 86.6%)
Examination	253	5.2	5.6 (4.8, 6.6)
Professional communication	1664	18.7	20.3 (19.0, 21.9)
Gather information	1678	27.1	29.4 (27.7, 31.5)
Documentation	551	16.3	17.7 (15.9, 19.6)
Unknown	11	0.7	0.8 (0.2, 1.6)
Social (both professional and non-professional)	507	6.1	6.7 (5.9, 7.6)
Hygiene	180	0.9	1.0 (0.8, 1.2)
Movement	925	5.4	5.9 (5.5, 6.3)
Outside ED	68	2.5	2.7 (1.8, 4.5)
Drug-related			17.8% (95% CI 16.8%, 19.3%)
Professional communication	504	5.5	5.9 (5.3, 6.6)
Gather information	491	6.5	7.0 (6.2, 7.9)
Documentation	376	6.1	6.6 (5.8, 7.5)

^1^Proportion of total observation time spent on task

^2^The proportions add up to more than 100% due to multitasking

Overall (both drug-related and non-drug-related) gathering information (36.5%), professional communication (26.3%) and documentation (24.2%) were the most time-consuming tasks. Gathering information was also the most time-consuming drug-related task (7.0% of total observation time, Table 2). 

When combining the most time-consuming task categories, with how tasks were conducted *(how)* and other personnel involved *(who)* (Table 3), gathering information on computer was the most time-consuming task combination overall, including both drug-related and non-drug-related (19.0% of total task time). Documentation on paper and computer was the most time-consuming drug-related tasks, 3.2% and 3.1% of total task time respectively (Table 3).

**Table 3:** With whom and how physicians conducted work tasks. Gather information, documentation, and professional communication (*what* with sub-categories *drug-related* vs. *non-drug-related,* combined with *who-* and *how)*. Highlighted cells (Italic) represent tasks included in the complex process of obtaining and documenting the patients’ drug lists

Task conducted with (WHO)	How task was conducted (HOW)	Drug-related tasks		Non-drug-related tasks	
		Number of recorded tasks	Proportion of time on task %^1, 2^ (95% CI)	Number of recorded tasks	Proportion of time on task %^1, 2^ (95% CI)
*Professional communication*					
Patient	Direct	84	1.08 (0.77, 1.56)	211	2.66 (2.09, 3.35)
Next of kin	Direct	19	0.25 (0.13, 0.40)	30	0.37 (0.19, 0.65)
Another physician	Direct/telephone	260	3.05 (2.64, 3.49)	730	9.82 (9.01, 10.65)
Nurse	Direct/telephone	119	1.07 (0.86, 1.35)	489	3.66 (3.30, 4.04)
Pharmacist	Direct	6	0.04 (0.01, 0.09)	-	-
Other hospital	Telephone	7	0.15 (0.06, 0.29)	16	0.68 (0.44, 1.00)
Unknown	Direct/telephone	15	0.40 (0.25, 0.62)	109	1.63 (1.30, 2.04)
Others	Direct/telephone	9	0.10 (0.05, 0.17)	82	1.59 (1.05, 2.43)
General Practitioner	Telephone	0	–	1	0.02
*Gather information*					
Patient	Direct	*200*	*2.57 (2.10, 3.11)*	400	8.42 (7.26, 9.53)
Next of kin	Direct	*22*	*0.21 (0.13, 0.34)*	53	0.65 (0.44, 0.93)
Another physician	Direct	1	0.01 (0.00, 0.02)	4	0.05 (0.01, 0.15)
Nurse	Direct	1	0.01 (0.00, 0.03)	3	0.02 (0.00, 0.07)
–	On paper	*57*	*1.08 (0.73, 1.58)*	253	2.91 (2.47, 3.37)
–	On computer	*153*	*1.80 (1.45, 2.20)*	961	17.22 (15.84, 18.66)
–	On smartphone	54	1.04 (0.76, 1.41)	12	0.21 (0.09, 0.40)
–	With Prescription Intermediary	*21*	*0.46 (0.26, 0.75)*	–	–
*Documentation*					
–	On paper	*144*	*3.19 (2.57, 3.83)*	83	0.67 (0.54, 0.83)
–	On computer	*186*	*3.09 (2.51, 3.67)*	400	15.12 (13.52, 17.03)
–	With dictaphone	*40*	*0.27 (0.16, 0.42)*	62	1.81 (1.28, 2.38)
–	With Prescription Intermediary	2	0.07 (0.02, 0.14)	–	–

^1^Proportion of total observation time spent on task

^2^Summarized proportion in this table exceeds proportions reported in Table 2 due to multitask

**Fig. 2:**
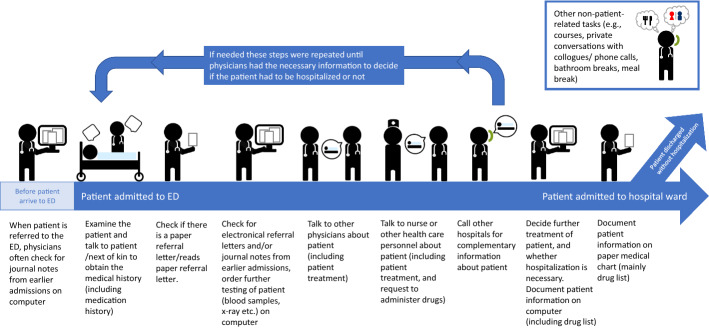
Illustration of physician tasks conducted during a typical emergency department (ED) visit (for one patient). Typically, the initial examination and communication with the patient were the most extensive, follow-up communication was more brief. Documentation in the electronic patient record was important for accessible information about the admission for healthcare personnel at hospital wards and documentation of the emergency department visit if patient was not hospitalized. Documentation on paper medical chart (mainly drug list) was used during the hospital stay e.g., by nurses at hospital wards to dispense drugs. Tasks present in the illustration is based on the collected data. Observation sessions were independent of the patient pathway. ED: emergency department

Obtaining and documenting a patient’s drug lists in the hospital systems was found to be a complex process consisting of a series of tasks (Table 3-highlighted cells (Italic)). This process occupied 12.9% (95% CI 11.9, 14.3) of ED physicians’ time, equivalent to 7.8 min (95% CI 7.2, 8.6) per hour on average. The process was fragmented through the patient’s stay in the ED (Fig. 2). Documentation on paper (medication chart) and computer/dictaphone (electronic patient journal) occupied approximately 4.0 min of the time spent on this process (documentation in both were required). An average of 1.7 min per hour was spent questioning the patient or next of kin about drugs, and an additional 2.0 min were spent gathering drug-related information on computer (including checking the Prescription Intermediary) or paper. 

Physicians were interrupted 368 times during the total observation time, which translates to an overall average interruption rate of 4.0 (95% CI 3.6, 4.4) times per hour. Interruption rate during drug-related task time were 4.3 (95% CI 3.0, 5.2) times per hour (*p* = 0.81, compared to the interruption rate for non-drug-related task time). The most interrupted drug-related task was documentation (55.6% of tasks with at least one interruption, during the time physicians conducted drug-related task). Professional communication was the most common reason for interruption of drug-related documentation (82.5% of interruptions).

Medical physicians spent more time than surgical physicians on drug-related tasks overall (19.1% (95%CI 17.5, 20.6) vs. 15.1% (95%CI 13.1, 17.2), *p* = 0.01), as well as for drug-related gathering information (7.7%, (95%CI 6.6, 8.8) vs. 5.4%, (95%CI 4.1, 6.9), *p* = 0.03) and drug-related professional communication (6.7% (95%CI 5.9, 7.4) vs. 4.5% (95%CI 3.7, 5.8), *p* = 0.01). There was no evidence of differences in time spent on any of the specific drug-related tasks, nor drug-related tasks overall between experienced and inexperienced physicians (17.8% (95%CI 16.0, 19.6) vs. 18.2 (95%CI 16.6, 20.0), *p* = 0.73).

## Discussion

### Statement of key findings

Among the nine conceptualized task categories gathering information, documentation, and professional communication were the most time-consuming for ED physicians in this study. ED physicians spend 17.8% of their time on drug-related tasks, and gathering information was the most time-consuming drug-related task. On average, physicians spent 7.8 min per hour on the complex process of obtaining and documenting patients’ drug lists. The ED physicians multitasked more during drug-related task time compared to non-drug-related tasks time. The overall interruption rate was 4.0 times per hour, there were no difference between drug-related task time and non-drug-related task time regarding interruption rates.

### Strengths and weaknesses

A validated method was used to perform the study [17] and high inter-rater agreement was achieved and maintained throughout the data collection period. WOMBAT utilized predefined categories. The discrete categories conceptualised and applied in this study were a compromise between the desire to collect as detailed data as possible and the practical feasibility of the study.

The number of observation hours (which is the sample size of concern in these kind of studies) are comparable to other time-motion studies aiming to describe work patterns [12, 18–20]. Due to the roster-based affiliation of physicians to the ED the proportion of physicians enrolled from the total number of available physicians were not calculated. However, observers randomized which of the available physicians to include and observed physicians with different affiliation, experience level, at different hours, and across a time-period of approximately 3 months. This provides the study with solid power regarding inter-individual variability. The study can therefore provide useful baseline information for future studies.

Data were collected between 9am and 9pm and may not be representable of night-time activities in the ED. However, the results represent the time distribution of physicians during the treatment of 80% of patients admitted to the investigated ED.

This study only involved one ED, thus potentially reduce the generalisability of the results. No other studies have investigating ED physicians’ drug-related task time. Hence, it is challenging to consider if the findings are representative of other EDs. Although, when looking at the results overall, they match many of the findings in a Danish ED study[12]. In Norway obtaining and documenting medication history is a physician task, hence the results from this study are not generalizable to EDs which have personnel specifically dedicated to obtaining medication history e.g., pharmacy technicians, pharmacists, or nurses.

### Interpretation

#### Physicians’ time distribution

ED physicians’ time distribution in the present study underlines the purpose of the ED and are similar to earlier studies [12, 18]. Gathering information is important to elucidate the patients’ presented symptoms, and to decide if the patient needs hospitalization. Documentation is important to inform the next level of care e.g., hospital ward or healthcare personnel in the primary healthcare. And professional communication with the patient and colleagues is essential among other to ensure safe and efficient treatment of the ED patient.

This is the first study of ED physicians quantifying time spent on all conducted drug-related tasks separately. Compared to physicians in hospital wards at an Australian hospital [25], physicians in the present study spent more time on drug-related tasks (7 vs. 17.8%, respectively). This is not surprising, normally a patient’s medication history is documented at admission to the ED. Hence, when the patient arrives at the hospital ward gathering of information and documentation of a patient’s drugs are already completed.

#### Obtaining and documenting patients’ drug lists

Gathering information about a patient’s medication history and current drug list is important as the drug list documented in the ED is used to decide further drug treatment during the hospital stay and after discharge.

The present study did not assess the quality of the obtained drug lists, it therefore remains undetermined whether the 7.8 min per hour (per patient as one new patient was assessed by physicians per hour on average) are sufficient to obtain a correct and complete drug list at admission. However, several prior studies have reported that ED drug lists frequently do not reflect the patients’ drug use prior to admission [3, 5, 8, 26]. According to the results from the present study physicians conduct numerous essential tasks during the patients ED stay, obtaining and documenting drug lists are only two of these tasks. This may explain that healthcare personnel in the ED dedicated to obtaining patients drug lists e.g., pharmacists, pharmacy technicians, document more accurate drug lists than physicians [2–4], as they focus on this specific task. Comparing the time spent by physicians obtaining and documenting drug lists in the present study with a systematic review on pharmacists conducting medication reconciliation in the ED setting, shows that the latter spend more time, reported 13.9-30 min per patient [8]. Further, it was also reported that medication discrepancies were reduced by 88% when ED pharmacists performed the medication reconciliation [8], indicating that the time spent and the systematic approach through medication reconciliation were worthwhile. As ED crowding is an increasing challenge, it should be considered to include healthcare personnel in the ED dedicated to obtaining patients drug lists. This can contribute to decrease ED physicians’ workload in the fast-paced workflow. However, the most important benefit is the potential decrease in medication discrepancies, especially for complex patients where the time spent by physicians may not be sufficient to obtain their complete and correct drug list.

#### Interruptions and multitasking

A German study reported that physicians were most frequently interrupted during documentation [27], and a Canadian study found that professional communication was the most common reason for interruption, which is in line with the results of the present study. In the present study interruption rates during drug-related and non-drug-related task time were equal. However, the frequency of multitasking was higher during drug-related task time compared to non-drug-related task time. According to an Australian study, multitasking and interruptions were associated with a higher rate of prescribing errors per medication order [13]. Interruptions and multitasking which result in prescribing errors at admission to ED can be a hazard against patient safety through the entire hospital stay and even after discharge [11].

#### Gather information in transition of care

Overall, the physicians in the present study spent approximately the same amount of time on documentation and communication as the ED physicians in a Danish study [12]. However, the physicians in the present study spent more time gathering information. The difference in study methods and definitions must be considered, although there may also be differences in accessibility of patient information between countries.

During the data collection period the only common electronic system between primary and secondary healthcare in Norway were the Prescription Intermediary, a nationwide electronic prescription database which includes information about patients’ prescribed drugs [28]. Another database has been implemented after the study were conducted, the “Summary care record”, which include a short summary of information needed in emergency care, e.g., information about critical adverse drug reactions and prescribed drugs. However, there are still no common patient record for primary and secondary healthcare with complete information about a patient’s medical and medication history. Physicians in the study had to use multiple sources to obtain this information. This can explain why no differences between drug-related time spent by experienced and inexperienced physicians were found, as checking multiple sources are equally time-consuming regardless of experience. The Prescription Intermediary was only checked for approximately every fourth patient. This was a surprising and noteworthy finding. Conclusions on why physicians did not take advantage of this easily accessible source cannot be drawn from the results. However, reliability can be a factor, as the database have to be manually updated the content in the Prescription Intermediary (and also the “Summary care record”) is not always trustworthy. With a short average length of stay in the ED, it is essential that electronic support tools are trustworthy to ensure that physicians’ limited time are used efficiently.

Medical physicians spent more time gathering drug-related information than surgical physicians. This may contribute to explain that earlier studies identified surgical admission/referral as a risk factor for clinically relevant medication discrepancies [2, 5].

#### Multidisciplinary interactions

Observed physicians in this study spent more time interacting (including both *professional communication* and *gathering information)* with other healthcare personnel than admitted patients, which is in line with another study[12]. Communication between colleague physicians and between physicians and nurses, are vital to ensure safe and efficient treatment of the ED patient. Although this cannot be quantified from the data (due to the discrete categories), it was noticed by the observers that some drug-related tasks were not conducted by physicians themselves but delegated to other health professions in the ED. Nurses were requested during *professional communication* with the observed physician to administer drugs to patients. And the secretary (*others*) was requested during *professional communication* to obtain information about patients’ drug lists from GP or nursing home.

Due to the limited pharmacist coverage in the investigated ED, it was not surprising that physicians only communicated with pharmacists 6 times during the data collection period. For instance, some observations sessions occurred when there was no pharmacist present. However, with adequate coordinating (e.g., a referral system or more resources), pharmacists could contribute to the process of obtaining and documenting correct and complete drug lists in the ED, as reported in prior studies [2, 3, 5].

#### Further research

The findings in this study raises some interesting questions regarding whether the time spent by ED physicians is sufficient to obtain a correct and complete drug list for admitted patients. And further, it could be explored if the interruptions during drug-related documentation could affect the quality of the drug lists. To answer this, future studies should combine time-motion observations with quality assessments of the obtained drug lists. In addition, further research could focus on how to optimize implementation of dedicated personnel to obtain drug-lists at admission e.g., pharmacists or pharmacy technicians, in the multidisciplinary team in the ED, and how this implementation could impact physicians’ time distribution.

## Conclusion

This is the first study to perform a detailed quantitative assessment of time spent on all drug-related tasks performed by ED physicians, in addition to the time distribution across other conducted tasks. Overall, 17.8% of ED physicians’ time was spent on drug-related tasks, and 7.8 min per hour (i.e., per patient) was spent on the complex process of obtaining and documenting the patients’ drug lists. This study adds important information that can be used for redesigning and optimising work- and information flow in transition of care when patients are admitted to the ED. In addition, it provides a useful baseline for future studies.

## Electronic Supplementary Material

Screenshot of the WOMBAT data collection tool with four dimensions. Below is the link to the electronic supplementary material 1
