# Supplementary material for: Emergency department physicians’ distribution of time in the fast paced-workflow-a novel time-motion study of drug-related activities
Source: Int J Clin Pharm. 2021 Dec 23;44(2):448–58. doi: 10.1007/s11096-021-01364-6 (PMC9007764; doi:10.1007/s11096-021-01364-6)
Supplement: Supplementary file 1 — Screenshot of the WOMBAT data collection tool with four dimensions. Below is the link to the electronic supplementary material 1 [file 11096_2021_1364_MOESM1_ESM.docx]

**Electronic supplementary material 1**

**Emergency department physicians’ distribution of time in the fast paced-workflow- a novel time-motion study of drug-related activities**

Lisbeth D. Nymoen^1,2^, Therese Tran^2^, Scott R. Walter^3^, Elin C. Lehnbom^4,5^, Ingrid K. Tunestveit^2^, Erik Øie^6^, Kirsten K. Viktil^1,2^

1 Diakonhjemmet Hospital Pharmacy, Oslo, Norway, 2 Department of Pharmacy, University of Oslo, Oslo, Norway, 3 Centre for Health Systems and Safety Research, Australian Institute of Health Innovation, Macquarie University, Sydney, Australia, 4 Department of Pharmacy, UiT The Arctic University of Norway, Tromsø, Norway, 5 Department of Health and Caring Sciences, Linnæus University, Kalmar, Sweden, 6 Department of Internal Medicine, Diakonhjemmet Hospital, Oslo, Norway

Corresponding author: Lisbeth Damlien Nymoen

Postal address: Diakonhjemmet Hospital Pharmacy AS, Postbox 40 Vinderen, 0319 Oslo, Norway

E-mail: lisbetd@student.matnat.uio.no

ORCID (corresponding author): 0000-0001-9330-3374

Journal: International Journal of Clinical Pharmacy

**Screenshot of the WOMBAT data collection tool with four dimensions.**

During direct observation of emergency department physicians, data were systematically registered using a Samsung Galaxy 8 tablet running version 2 of the licenced WOMBAT software(1, 2). Data were registered in predefined discrete categories organised under four dimensions: *WHAT, WHERE, HOW, AND WHO*. And were automatically time-stamped by the WOMBAT software i.e., recording the exact time for the start of the task and recording the time until a new task was started (either due to finishing the task or getting interrupted).


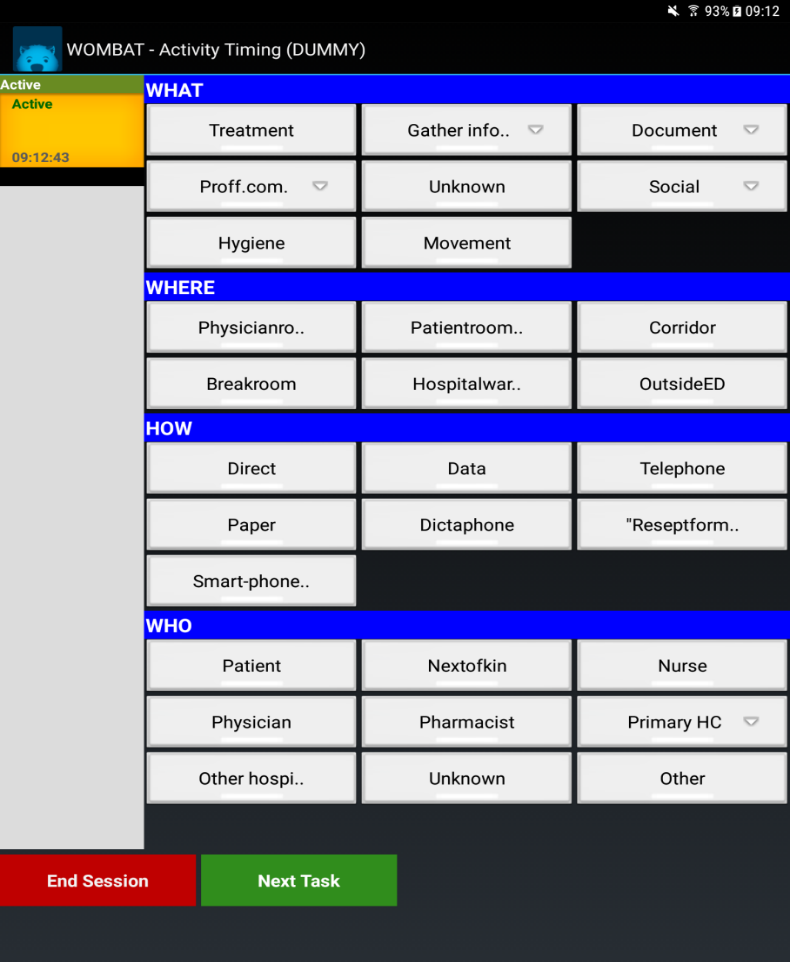


***WHAT***

The *WHAT* dimension described the work tasks conducted by the observed emergency physician. This dimension was mandatory. Categories, sub-categories, definitions, and examples of work tasks, *WHAT* dimension is presented in Table 1 in the article.

Explanation of truncated words in the figure:

Treatment; treatment/examination,

Gather info.; gather information

Document; documentation

Proff.com; professional communication

***WHERE***

The *WHERE* dimension was utilized to record where the observed emergency physician conducted the recorded task. This dimension was mandatory (connected to every recorded work task, *WHAT*).

**Physician room** (Physicianro)**:** the office of the emergency department physicians

**Patient room** (Patientroom)**:** rooms dedicated to patients admitted to the emergency department

**Corridor:** the corridor in the emergency department, leading to physician room and all patient rooms

**Break room** (Breakroom)**:** room for meal breaks and socialising

**Hospital ward** (Hospitalwar.)**:** hospital wards at Diakonhjemmet Hospital

**Outside emergency department** (OutsideED)**:** when emergency department physicians left the emergency department for whatever reason

As the aim of this study was to investigate emergency department activities, work tasks recorded as performed at hospital wards and outside emergency department were merged to one work task category, under the *WHAT* dimension called *outside emergency department* see article.

***HOW***

The *HOW* dimension was utilized to record how the observed emergency physician conducted the recorded task. This dimension was not mandatory as some work tasks did not require this dimension, e.g., hygiene, movement. However, this dimension was recorded connected to recorded work task, *WHAT* whenever appropriate.

**Direct:** face-to-face interaction

**Data:** reading or writing on computer

**Telephone:** talking on the work telephone, each emergency department physician on call have a work phone available

**Paper:** reading or writing on paper

**Dictaphone:** recording via dictaphone information which was transcribed to text by transcribers employed at Diakonhjemmet Hospital, some of the emergency department physicians utilized this instead of writing the admission note in the electronically patient record

**The prescription intermediary** (Reseptform.)**:** a nationwide electronic prescription database which is available both to primary and secondary healthcare and contain information about patients’ prescribed drugs

**Smart phone:** checking apps for professional information, talking on the telephone, answering private texts

***WHO***

The *WHO* dimension was utilized to record with whom the observed emergency physician conducted the recorded task. This dimension was not mandatory as some work tasks did not require this dimension, e.g., documentation, hygiene, movement. However, this dimension was recorded connected to recorded work task, *WHAT* whenever appropriate.

**Patient:** observed physician interacted with a patient

**Next of kin:** observed physician interacted with next of kin of an admitted patient

**Nurse:** observed physician interacted with nurse

**Physician:** observed physician interacted with another physician

**Pharmacist:** observed physician interacted with a pharmacist

**Primary healthcare service** (Primary HC)**:** observed physician interacted with healthcare personnel in the primary healthcare service, general practitioner (GP), nursing home, municipal emergency clinic

**Other hospitals** (Other hospi.)**:** observed physician interacted with healthcare personnel at another hospital

**Unknown:** observed physician interacted with someone which could not be identified by the observers

**Other:** observed physician interacted with other persons which were not covered by the pre-set categories e.g., emergency department secretary, personnel from the hospital laboratory

***Examples of data collection:***

| **Observation** | **WHAT** | **WHERE** | **HOW** | **WHO** |
| --- | --- | --- | --- | --- |
| Physician taking medication history with the patient in the patient room | Gather information, drug-related (subcategory) | Patient room | Direct | Patient |
| Physician writing background information about a patient’s medical history on computer in the physician office | Documentation,  non-drug-related (subcategory) | Physician room | Data | - |
| Physician examining the patient in the patient room | Treatment/Examination | Patient room | Direct | Patient |
| Physician discussing the patients drug treatment with a colleague physician and a nurse in the physician office | Professional communication,  drug-related  (subcategory) | Physician room | Direct | Physician & Nurse |
| Physician writes patients drug list, and further drug-treatment for the hospital stay on the hospital medication chart while in the physician office | Documentation,  drug-related (subcategory) | Physician room | Paper | - |
| Physician walks from patient room to physician office | Movement | Corridor | - | - |
| Physician asks nurse (in the corridor) to administer a drug to a patient | Professional communication,  drug-related (subcategory) | Corridor | Direct | Nurse |
| Physician asks secretary (in the physician room) to obtain drug-list from the patients GP | Professional communication,  drug-related (subcategory) | Physician room | Direct | Other |

**REFERENCES**

1. Westbrook JI, Ampt A. Design, application and testing of the Work Observation Method by Activity Timing (WOMBAT) to measure clinicians' patterns of work and communication. Int J Med Inform. 2009;78 Suppl 1:S25-33.

2. Ballermann MA, Shaw NT, Mayes DC, Gibney RT, Westbrook JI. Validation of the Work Observation Method By Activity Timing (WOMBAT) method of conducting time-motion observations in critical care settings: an observational study. BMC Med Inform Decis Mak. 2011;11:32.
